# Antimicrobial, Multi-Drug and Colistin Resistance in *Enterobacteriaceae* in Healthy Pigs in the Greater Accra Region of Ghana, 2022: A Cross-Sectional Study

**DOI:** 10.3390/ijerph191610449

**Published:** 2022-08-22

**Authors:** Rita Ohene Larbi, Wisdom Adeapena, Matilda Ayim-Akonor, Ebenezer D. O. Ansa, Hannock Tweya, Robert Fraser Terry, Appiah-Korang Labi, Anthony D. Harries

**Affiliations:** 1 CSIR—Animal Research Institute, P.O. Box AH 20 Achimota-Accra, Greater Accra Region, Ghana; 2 Kintampo Health Research Centre, P.O. Box 200, Kintampo, Bono East Region, Ghana; 3 Malawi International Training and Education Center for Health (Malawi-I-TECH), Lilongwe P.O. Box 30369, Malawi; 4 Special Programme for Research and Training in Tropical Diseases, World Health Organization, 1211 Geneva, Switzerland; 5 WHO Country Office, P.O. Box MB 142, 7 Ameda Street, Roman Ridge-Accra, Greater Accra Region, Ghana; 6 Centre for Operational Research, International Union Against Tuberculosis and Lung Disease (The Union), 75001 Paris, France; 7 Department of Clinical Research, Faculty of Infectious and Tropical Diseases, London School of Hygiene and Tropical Medicine, London WC1E 7HT, UK

**Keywords:** farms in Ghana, antimicrobial use, *Escherichia coli*, *Klebsiella pneumoniae*, *Enterobacter* spp., antimicrobial resistance (AMR), *mcr-1* gene, operational research, SORT IT

## Abstract

There is little published information on antimicrobial resistance (AMR) in animals in Ghana. We determined the prevalence and factors associated with AMR, multi-drug resistance (MDR-resistance to ≥3 antimicrobial classes) and colistin resistance in *Enterobacteriaceae* in healthy pigs in Accra, Ghana. Rectal swabs obtained from the pigs on 20 farms from January to March 2022, were examined for *Escherichia coli*, *Enterobacter* spp. and *Klebsiella pneumoniae*. AMR was determined using standard microbiological techniques and the *mcr-1* gene detected through molecular analysis. *Enterobacteriaceae* were isolated from 197 of 200 pigs: these comprised 195 *E. coli* isolates, 38 *Enterobacter* spp. and 3 *K. pneumoniae*, either singly or combined. Over 60% of *E. coli* were resistant to tetracycline, with 27% and 34% being resistant to amoxicillin/clavulanic acid and ampicillin, respectively; 23% of *E. coli* and 5% of *Enterobacter* spp. exhibited MDR phenotypes. Phenotypic colistin resistance was found in 8% of *E. coli* and *Enterobacter* spp., with the *mcr-1* gene detected in half. Our study findings should be incorporated into on-going AMR, MDR and colistin resistance surveillance programs in Ghana. We further advocate for tailored-specific education for pig farmers on animal antimicrobial use and for strengthened regulatory policy on antimicrobial usage and monitoring in the animal production industry.

## 1. Introduction

Antimicrobial resistance (AMR) is a complex and multifaceted issue that is recognized as one of the most serious global threats to human health in the 21st century [1]. There are several factors contributing to this menace which include use and misuse of antimicrobial agents in humans and animals, the movement of human populations across the globe influencing transmission and exposure of people to pathogenic and antimicrobial-resistant strains of bacteria [2].

The emergence of multi-drug resistance (MDR) is a particularly concerning development. MDR is defined as resistance to at least one agent in three or more antimicrobial classes [3] and is particularly found among *Enterobacteriaceae* such as *Escherichia coli* (*E. coli*) and *Klebsiella pneumoniae* (*K. pneumoniae*). Both organisms cause serious infections and have multiple resistance mechanisms, the most common being extended-spectrum β-lactamase (ESBL) and carbapenemase production. In recent times, there has been a dramatic increase in MDR-*Enterobacteriaceae* in animals, and this may constitute both an indirect risk to human public health by increasing the gene pool from which pathogenic bacteria can pick up resistant genes and a direct risk through the food chain [4].

High levels of MDR, including ESBL, among *Enterobacteriaceae* have been found in pigs and pork meat in Nigeria, Cameroon, Tanzania, Uganda and South Africa, raising concerns about transmission to humans via the food chain [5,6,7,8,9]. However, a recent review of ESBL and carbapenemase-producing *Enterobacteriaceae* in West and Central Africa revealed that only 20 studies focusing on animals had been published between 2000 and 2020 [10]. This review concluded that there were major knowledge gaps about MDR and ESBL-producing *Enterobacteriaceae* in animals in Africa that would limit AMR management in these regions unless the evidence base was expanded [10].

Colistin (or polymyxin E) is an antibiotic that is considered a last resort therapeutic option in humans against MDR Gram-negative bacterial infections. Colistin has been widely used in animal husbandry for the last three decades for the treatment and prevention of *Enterobacteriaceae* infection as well as for growth promotion in animal feeds [11], and this has particularly been the case in the pig production industry [12]. Colistin resistance may be mediated through chromosomal mutation or transferred between bacteria by plasmid borne genes [12]. A plasmid borne *mcr-1* gene encoding for colistin resistance was first identified in pigs and humans in China in 2014 [13]. Since then, colistin resistance has been identified across the six continents of the world in human, animal and environmental samples with ten genes now being associated with it (*mcr-1 to mcr-10*) [14].

Colistin has been used in animal farming on the African continent for years, but to date there is limited information on colistin resistance in African countries. A recent study in Nigeria found that 17% of bacterial isolates from human and animals, including pigs, were resistant to colistin with resistant strains often associated with the *mcr-1* gene [15]. There is growing concern that the African pig rearing industry may be a reservoir for colistin-resistant bacteria [16]. In Ghana, there are no specific restrictions on colistin use in animals, and there is no published information about colistin use in the pig industry, either for treatment or as a growth promoter. Three studies published in 2012, 2014 and 2021 on drug resistance in *E. coli* isolates from poultry, pigs and cattle in Ghana [17,18,19] demonstrated high levels of MDR, raising concerns about food animals being an important reservoir for AMR in the country. No studies could be identified on the presence of colistin resistance in animals in Ghana. There have been calls to improve the evidence base not only on MDR in *Enterobacteriaceae* but also on colistin use, colistin resistance and associated risk factors among animals and particularly pigs [6,20].

We therefore conducted a study to determine the prevalence of AMR, MDR and colistin-resistant *Enterobacteriaceae* in healthy pigs in Ghana and assess factors associated with these types of AMR with a view to generating relevant baseline data to better inform the AMR dynamics in the country. We concentrated on three bacteria belonging to the family *Enterobacteriaceae* (*E. coli*, *K. pneumoniae* and *Enterobacter* spp.) because these three bacteria are important causes of human and animal infections. The specific objectives were to determine in healthy pigs in selected farms in the Greater Accra region of Ghana between January and March, 2022: (i) the characteristics of the pigs and their farms as well as the types of antimicrobials used; (ii) number and proportion of rectal swabs growing *Enterobacteriaceae*; (iii) number and proportion of *Enterobacteriaceae* isolates with AMR, MDR and colistin resistance; and (iv) antimicrobial use associated with AMR, and pig and farm characteristics associated with MDR and colistin resistance.

## 2. Materials and Methods

### 2.1. Study Design

This was a cross-sectional study using primary data.

### 2.2. Setting and Investigative Procedures

#### 2.2.1. General Setting

Ghana is a country located along the Gulf of Guinea and Atlantic Ocean, in the sub-region of West Africa. Ghana has a population of approximately 30.8 million and Accra is the capital city with a population of 5.4 million [21].

#### 2.2.2. Site Specific Setting

The Greater Accra region of Ghana comprises 29 districts [21]. Based on information obtained from the Greater Accra Pig Farmers Association and the Ministry of Food and Agriculture, four districts with a high concentration of commercial pig farms were selected. Within each district, five pig farms were randomly selected. The four districts comprised: *Adenta*—a population size of 237,546 and 7% of households engaged in agriculture, mainly crop farming and livestock rearing that includes chickens, goats, sheep and pigs; *Ningo Prampram*—a population size of 204,673 and 44% of households engaged in livestock rearing of chickens, goats, cattle, sheep and pigs; *Shai Osudoku*—a population size of 105,610 and 50% of households engaged in livestock rearing of chickens, guinea fowl, turkeys, goats, cattle and pigs; *Ga South*—a population size of 350,121 and 12% of households engaged in agriculture and livestock rearing, especially chickens, goats and pigs [21,22].

#### 2.2.3. Sample Collection

At each of the twenty selected farms, informed consent (using signature or thumbprint) about participating in the study was obtained from the farm owners, who were also given an explanatory leaflet about the study. After this, a questionnaire was administered by a trained field investigator to collect information about the farm and the pigs. In instances where farmers did not understand English, the questionnaire was interpreted into a local language, in a manner which did not distort the meaning of the questions.

Ten healthy pigs that had gone past the weaning stage but were aged less than 12 months and whose weights averaged between 20 kg and 50 kg were selected based on sampling methodology related to the number of pens that housed this type of pigs on each farm. The number of pens varied from 3–10 per farm. Pigs were randomly selected from between 3–4 pens on each farm, and on no farm were pigs selected just from one pen.

Apparently healthy rather than unhealthy pigs were studied because they are the principal source for human consumption [23]. Unhealthy pigs were identified with the help of a veterinarian and were not included in the study. One rectal swab per pig was taken by the veterinarian using a sterile cotton swab stick, with the swabs taken between January and March 2022. The rectal swabs were kept in 1 mL buffered peptone water and transported on ice to the microbiology laboratory of the CSIR-Animal Research Institute for further processing. All swabs were processed within 24 h of collection.

#### 2.2.4. Specimen Processing and Laboratory Procedures

At the laboratory, each rectal swab was incubated in a final volume of 9 mL buffered peptone water for 18–24 h at 37 °C, and all measures were taken to prevent contamination. A loopful of this culture was streaked out on MacConkey agar (Becton, Dickson and Company, Franklin Lakes, NJ, USA) and incubated at 37 °C for 24 h. Colonies showing distinct bacterial morphology of *E. coli, K. pneumoniae* and *Enterobacter* spp. were plated out on Eosin Methylene Blue agar (Oxoid Ltd., Hampshire, UK) and incubated under same conditions. Standard biochemical tests (which included Simmons Citrate, Triple Sugar Iron, Urea, Indole Sulphide and Methyl Red) were performed to further confirm the isolates [24]. Antimicrobial susceptibility testing was carried out on all confirmed isolates using the Kirby Bauer disc diffusion method with the following antimicrobial discs (Oxoid Ltd., Hampshire, UK): ampicillin (10 µg), amoxicillin/clavulanic acid (30 µg), ceftazidime (30 µg), ciprofloxacin (5 µg), gentamicin (10 µg), tetracycline (30 µg), trimethoprim-sulfamethoxazole (25 µg), chloramphenicol (30 µg) and aztreonam (30 µg). In bacteria that had intrinsic resistance to a specific antimicrobial, testing was not carried out. The nine antimicrobials belong to different classes. Six of the antimicrobials (ampicillin, amoxicillin/clavulanic acid, gentamicin, ciprofloxacin, tetracycline and trimethoprim-sulfamethoxazole) are classified as veterinary critically important antimicrobial agents (VCIA) by the World Organisation for Animal Health [25] and three others (aztreonam, ceftazidime and chloramphenicol) are considered highly important antimicrobials in human health [26]. The reference strain *E. coli ATCC* 25922 was used as a quality control. The Clinical Laboratory Standards Institute (CLSI) guidelines were used in interpreting the results of antimicrobial susceptibility testing [27]. Resistance to three or more antimicrobial classes was classified as MDR [3].

All confirmed isolates were also plated on Chromagar COL-APSE (CHROMagar, Paris, France) to screen for presumptive phenotypic colistin resistance using the protocol described by Momin and others [28]. For molecular analysis of the *mcr-1* gene, DNA was extracted from phenotypic colistin-resistant isolates by the boiling method and the primers CLR5-F (5′CGGTCAGTCCGTTTGTTC-3′) and CLR5-R (5′-CTTGGTCGGTCTGTA GGG-3′) were used [7,8]. Total reaction volume was set as 25 µL by adding 22 µL of 1× master mix, 0.5 µL each of forward and reverse primer and 2 µL of DNA template. The optimized polymerase chain reaction (PCR) amplification cycle of *mcr-1* gene was 95 °C for 5 min; 35 cycles of denaturation at 95 °C for 30 s; annealing at 53 °C for 30 s; extension at 72 °C for 30 s, final extension at 72 °C for 10 min and holding at 4 °C for infinity. Amplicons (with expected fragment size of 309 bp) were resolved on a 1.5% ethidium bromide-stained agarose gel and visualized with a UV transilluminator (Gel Doc).

### 2.3. Study Population

The study population included ten pigs from each of the 20 selected farms: in total, 200 pigs. Based on two previous studies in Ghana looking at MDR in *E. coli* [18,19], we estimated an MDR prevalence in *Enterobacteriaceae* of about 85%. Using the free software package “Open Epi”, we calculated a sample size of 196 based on population size of pigs, an estimated prevalence of 85%, 95% confidence intervals and a design effect of 1.0 for a random sample. Hence, our decision to go for a total sample size of 200 pigs.

### 2.4. Data Variables and Sources of Data

Data variables from the pigs and farms were: age of the pig; sex of the pig; breed of pig (exotic = imported breeds, local = breeds from Ghana); annual production turnover on the farm; farm feed (commercial, self-made, both); antimicrobials added to feed (for example, colistin and tetracycline); antimicrobials used on the farm as treatment and/or prophylaxis. Microbiological data variables included: *Enterobacteriaceae* isolated from rectal swabs and identified as *E. coli*, *K. pneumoniae* and *Enterobacter* spp.; for each bacterial isolate, antimicrobial susceptibility or resistance (defined as intermediate or resistant) to ampicillin, amoxicillin/clavulanic acid, ceftazidime, ciprofloxacin, gentamicin, tetracycline, trimethoprim-sulfamethoxazole, chloramphenicol and aztreonam. MDR was defined as resistance to three or more of the antimicrobial classes; in this definition, we excluded colistin. For colistin, the results were categorized as sensitive or resistant, and for those showing phenotypic resistance whether the *mcr-1* gene was detected or not.

### 2.5. Data Management, Analysis and Statistics

Data on the pigs and farms were collected using Open Data Kit (ODK)—this is a free open-source application allowing researchers to create a questionnaire form and complete it on a mobile phone or tablet without the need for internet [29]. Through ID numbers, the ten pigs on each farm were linked to the characteristics of that farm and in turn each pig was linked to the laboratory data generated at the CSIR Animal Research Institute Laboratory.

The data from ODK was extracted into MS Excel, and subsequently exported to EpiData Analysis version 2.2.2.186 (EpiData Association, Odense, Denmark) and Stata v13 (Stata Corporation College Station, College Station, TX, USA) for further cleaning and analysis. A descriptive analysis was performed to determine frequencies and proportions from categorical variables such as characteristics of the pigs and the farms in which they were reared, isolates of *Enterobacteriaceae* (stratified by *E. coli*, *K. pneumoniae* and *Enterobacter* spp.) and isolates showing antimicrobial resistance, MDR and colistin resistance (with and without the *mcr-1* gene). Pig and farm characteristics associated with AMR, MDR or colistin resistance were assessed using the chi-square test and results in the tables presented as Prevalence Ratios with 95% Confidence intervals (95% CI)—the calculation of prevalence ratios took clustering into effect. Levels of significance were set at 5% (*p* < 0.05).

## 3. Results

### 3.1. Characteristics of the Pigs and Their Farms as Well as the Types of Antimicrobials Used

There were 200 apparently healthy pigs from 20 selected farms that were included in the study, the characteristics of which are shown in Table 1. There were slightly more males (n = 104) compared with females (n = 96), their median age was 4 months, and they were all exotic breeds. All the pigs in the 20 farms were housed in pens. There was an annual production turnover between 30 and 100 pigs in 40% of farms, between 101 and 300 pigs in 40% of farms and more than 300 pigs in the remainder. Three-quarters of the farms fed their pigs with self-made feed. The remaining quarter either used commercial feed or combined it with self-made feed. In the previous 12 months, farmers reported the use of antimicrobials as treatment and/or prophylaxis in all the 20 farms. In all the 20 farms, farmers reported that antimicrobials were not used as growth promoters in farm feed.

The types of antimicrobials (according to their common active ingredients) used on the 20 pig farms, along with their respective WHO AWaRe (Access, Watch and Reserve Antimicrobials) classifications, are shown in Table 2. Overall, there were 36 different brands of antimicrobials used, containing 13 common active ingredients. Of these active ingredients, 7 (54%) were in the WHO Access category, 3 (23%) were in the Watch category, 1 (8%) was in the Reserve and 2 (15%) were not classified because they are veterinary drugs. Over half of the farms used streptomycin/gentamicin and penicillin/amoxicillin, with the next three most common active ingredients being the tetracycline class (45%), sulphonamides/trimethoprim (30%) and the macrolide class-tylosin tartrate/erythromycin (25%). Colistin sulphate was used on one farm only. One farm stated that they used herbal treatment for their pigs.

### 3.2. Enterobacteriaceae Isolated from Pig Rectal Swabs

Numbers and proportions of *Enterobacteriaceae*, stratified by *E. coli*, *K. pneumoniae* and *Enterobacter* spp. are shown in Table 3. All but 3 rectal swabs yielded at least one *Enterobacteriaceae* isolate. Altogether, there were 195 isolates of *E. coli*, 3 isolates of *K. pneumoniae* and 38 of *Enterobacter* spp. In 159 (81%) rectal swabs, only one species of *Enterobacteriaceae* was found while in the other 38 (19%) swabs, there were combinations of 2 or 3 *Enterobactericeae*.

### 3.3. Enterobacteriaceae Showing AMR, MDR and Colistin Resistance

**Figure 1:**
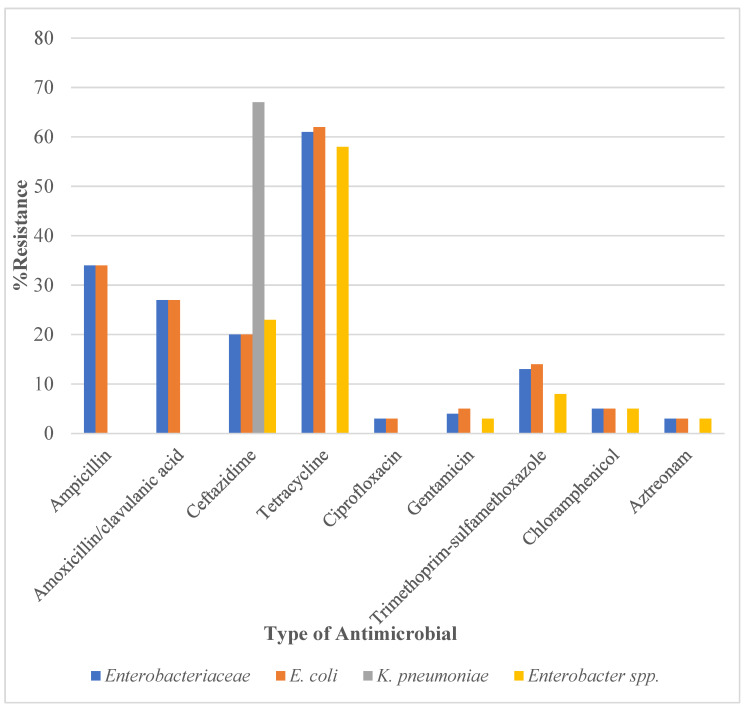
Antimicrobial resistance in *Enterobacteriaceae* isolated from healthy pigs in the Greater Accra Region of Ghana between January and March 2022. Footnote: No data on resistance for ampicillin and amoxicillin/clavulanic acid in *Enterobacter* spp. and *K. pneumoniae* due to intrinsic resistance. Resistance includes all isolates that were not susceptible to the antimicrobial (i.e., intermediate + resistant). Note that the 66% resistance to ceftazidime applies to only three *K. pneumoniae* isolates.

The distribution of AMR in *Enterobacteriaceae* is shown in Figure 1. Over 60% of *Enterobactericeae* were resistant to tetracycline and similar results were found for *E. coli* and *Enterobacter* spp. Between 27% and 34% of *Enterobacteriaceae* were resistant to ampicillin and amoxicillin/clavulanic acid. Between 10% and 20% were resistant to ceftazidime and trimethoprim-sulphamethoxazole. For the other antimicrobials, the prevalence of resistance was 5% or less, with the least resistance being found to ciprofloxacin and aztreonam.

The prevalence of MDR for the three types of *Enterobacteriaceae* is shown in Table 4. Altogether, 44 (23%, 95% CI 17–29) of 195 *E. coli* isolates were MDR. Resistance to three antimicrobial classes was found in 18 (9%) isolates, to four antimicrobial classes in 16 (8%), to five antimicrobial classes in 9 (5%) and to six antimicrobial classes in 1 (0.5%). Altogether, 2 (5%, 95% CI 1–16) of 38 *Enterobacter* spp. Isolates were MDR. Resistance to three antimicrobial classes was found in 1 (3%) isolate and to four antimicrobial classes in 1 (3%). For *K. pneumoniae*, no MDR was detected.

There was variation in the MDR profile of the 46 bacterial isolates as shown in Table 5. There were 9 different antibiotic combinations when resistance was found in three antimicrobial classes, 10 different combinations in four classes, 6 different combinations in five classes and 1 combination in six classes.

The prevalence of colistin resistance in *Enterobacteriaceae* including *E. coli* and *Enterobacter* spp. is shown in Table 6. For all 236 *Enterobacteriaceae* isolates, phenotypic colistin resistance was found in 18 (7.6%), with similar results for *E. coli* and *Enterobacter* spp. For those *Enterobacteriaceae* with colistin resistance, the *mcr-1* gene was detected in 10 (56%): the prevalence of *mcr-1* gene was found in 60% of *E. coli* and 33% of *Enterobacter* spp., respectively. None of the three isolates of *K. pneumoniae* showed phenotypic colistin resistance.

### 3.4. Factors associated with AMR, MDR and Colistin Resistance

In farms that had data on specific antimicrobial use, associations were assessed between use of antimicrobials on the farms and antimicrobial resistance in the pigs for tetracycline and ampicillin. Tetracycline resistance was found in 59/106 (56%) *Enterobacteriaceae* in pigs reared on farms using tetracyclines which was not significantly different from 84/132 (64%) tetracycline-resistant *Enterobacteriaceae* in pigs reared on farms which did not use tetracyclines, *p* = 0.21. Similarly, ampicillin resistance was found in 39/131 (30%) *Enterobacteriaceae* in pigs reared on farms using penicillins which was not significantly different from 22/80 (28%) ampicillin-resistant *Enterobacteriaceae* in pigs reared on farms which did not use these antibiotics, *p* = 0.72.

Factors associated with MDR and colistin resistance in healthy pigs are shown in Table 7 and Table 8, respectively. For pig and farm characteristics, no significant associations were found for MDR or for colistin resistance. In particular, on the one farm that used colistin, no pigs were found with colistin resistance.

## 4. Discussion

This was the first study conducted in the Greater Accra Region of Ghana in healthy pigs assessing the prevalence of *Enterobacteriaceae* (*E. coli*, *Enterobacter* spp. and *K. pneumoniae*), the pattern and distribution of AMR, MDR and colistin resistance and associated factors. There were four key findings.

First, *E. coli* was identified as the dominant species in pig rectal swabs in this study, followed by *Enterobacter* spp. and *K. pneumoniae.* These findings are consistent with previous studies that have examined the fecal flora of healthy pigs [11,18,30]. *E. coli* is one of the most common colonizers of the gastrointestinal tract in both animals and humans, and this confirms the use of this bacterium as an indicator for estimating the burden of AMR in animals [23]. Conversely, while *K. pneumoniae* naturally colonizes the respiratory and gastrointestinal tract of humans, its low prevalence in animals might be due to diet, competition with other bacteria in the environment or genetic virulence factors responsible for colonization of enteric systems in animals [31].

Second, the predominant findings with respect to AMR were that nearly two thirds of *Enterobacteriaceae* were resistant to tetracycline and nearly one third were resistant to ampicillin and amoxicillin/clavulanic acid. Levels of resistance to the other tested antimicrobials were fairly minimal, with most being less than 10%. The high levels of AMR to tetracycline and ampicillin have been reported previously in Ghana [18,19] as well as in other African countries such as Uganda and Tanzania [11,32]. The high AMR levels in Ghana may be due to the predominant use of tetracycline in animal husbandry in the country [33].

On all the 20 farms, the farmers stated that antimicrobials used in the previous 12 months were mainly veterinary drugs and that they were used for therapeutic purposes and occasionally for prophylaxis. This is consistent with what was found in the Ashanti region of Ghana where antimicrobials were mainly used for treatment, the main antimicrobial classes being aminoglycosides, penicillins and tetracyclines [34]. We found no association between the farm use of tetracyclines and penicillins as treatment and/or prophylaxis and their corresponding AMR in the *Enterobacteriaceae*. While this may seem surprising, tetracyclines and penicillins have been used for several decades in livestock production and resistance may build up on a farm over many years. Few farmers disinfect their entire farmhouses and dust samples and fomites may be a good source of antimicrobial-resistant genes [35,36]. Thus, even though animals present on a farm may not have been given certain antimicrobials, AMR bacteria can still be acquired. The lack of proper biosecurity measures such as restricting movement of people on the farms and preventing animals such as domestic dogs, chickens and wild birds from straying onto farms could be another factor which facilitates the exposure of the pigs to environmental transmission of AMR [37,38].

Third, we found a relatively lower MDR prevalence of 23% for *E. coli* compared with two previous studies in the Ashanti region of Ghana that had recorded a prevalence of 42% and 96%, respectively [18,19]. We identified no factors amongst the pigs or the farms that were associated with MDR, which is similar to findings from small-holder pig farms in Uganda [12]. However, in contrast, in Tanzania the presence of exotic breeds and recent antimicrobial use significantly predicted colonization with MDR *E. coli* [39]. The encouraging lower MDR prevalence in our study compared with the other two studies in Ghana studies could be due to the different geographical locations and/or different farm practices in the two regions. From our questionnaire study, farmers tended to treat individual animals presenting with symptoms rather than treating the entire herd of pigs. A study in Cambodia investigating MDR in pigs observed that there was a higher prevalence of MDR in *E. coli* from farms that treated entire herds and from farms that administered frequent prophylaxis [40].

Fourth, phenotypic colistin resistance was identified in about 8% of *E. coli* and *Enterobacter* spp., and we could find no factors associated with this. In particular, the one farm that used colistin had no pigs identified with colistin resistance. In the *E. coli,* 60% of phenotypic positive isolates were associated with the *mcr-1* gene. This suggests that while some colistin resistance can be mediated through chromosomal mutation, over half of the resistance was due to plasmid-mediated transfer of the *mcr-1* gene. A recent review found a global prevalence of colistin resistance of 1.6%, although incidence rates have risen over the last five years in all continents, largely as a result of inappropriate use of the antimicrobial in animal husbandry [41]. Since the *mcr-1* gene was first detected in pigs and humans in China in 2014 [7], there has been rapid and concerning world-wide spread of the gene [42]. Thus, while colistin resistance prevalence was relatively low in our study, this will need regular monitoring in the future in Ghana.

The strengths of this study were the large sample size and robust microbiology procedures to identify AMR and colistin resistance. The conduct and reporting of the study were also in line with the STROBE statement (Strengthening the Reporting of Observational Studies in Epidemiology [43]).

However, there were some limitations. There were some missing data from the farms. There was no record as to which of the individual pigs were given antimicrobials for treatment and the frequency of antibiotic administration, either for treatment or prophylaxis, was not recorded. Due to resource limitations, in bacteria with phenotypic colistin resistance, we did not investigate whether the other nine variants of the *mcr*-gene (*mcr 2 –mcr 10*) were present. We also did not determine whether the *mcr-1* gene was present in *Enterobacteriaceae* that were phenotypically sensitive to colistin, which would have alerted us to the possible presence of molecular resistance that has yet to translate into phenotypic and clinically relevant resistance.

There are three important implications and recommendations from this study. First, our study findings could be integrated into on-going surveillance of AMR, MDR and colistin resistance amongst pigs and other farm animals such as poultry, and our study methodology could be replicated in other regions of the country. Part of this surveillance should include more detailed research. For example, in our study, three quarters of the farm feed was self-made with the other quarter consisting of commercial feed. Despite farmers’ statements that antimicrobials were not added to farm feeds, these feeds need to be tested for antimicrobial residues to ensure they do not contain active antimicrobial ingredients which can lead to the spread of AMR in the country. We could also examine through a One Health approach other environmental samples from farms, such as dust and water, for bacteria and AMR.

Second, although farmers were not specifically asked about their knowledge of antimicrobial usage, we recommend that this is assessed more thoroughly and if found deficient, remedied through public health education. Farmers should also be entreated to seek the advice of veterinarians on how to properly use antimicrobials. Relevant stakeholders need to be actively involved in implementing this.

Third, the Ghana National Action Plan on Antimicrobial Resistance 2017–2021 promotes the responsible use and monitoring of antimicrobials in animals [44]. However, there is no mention about the use of antimicrobials as growth promoters. In line with this plan, we call for regulatory policy in Ghana to be strengthened with respect to the use of antimicrobials as growth promoters in the animal industry. An encouraging report from China showed that banning colistin as an animal growth promoter led to significant decreases in colistin-resistant *E. coli* in pigs, chicken and human [45]. Strong regulatory policy combined with AMR monitoring can work, and this should help to prevent the spread of AMR, MDR and colistin resistance in Ghana.

## 5. Conclusions

In conclusion, *E. coli* was the predominant bacteria identified in 200 healthy pigs. Amongst the *E. coli* isolates, 60% showed resistance to tetracycline and 27–34% showed resistance to penicillins; 23% showed MDR and approximately 8% showed phenotypic colistin resistance. We identified no factors in the pigs, the farms or related to antimicrobial use that correlated with these microbiological findings. We recommend the incorporation of our study findings into on-going AMR, MDR and colistin resistance surveillance programs in Ghana and further advocate for tailored, specific education for pig farmers on antimicrobial use in their animals. In addition, regulatory policy on antimicrobial usage and monitoring in the animal industry should be strengthened.

## Figures and Tables

**Table 1:** Characteristics of healthy pigs and the farms in which they were reared in the Greater Accra Region of Ghana between January and March 2022.

Characteristics		Number	(%)
**Pigs (Total)**		200	
Sex	Male	104	(52)
	Female	96	(48)
Age in months	Median (IQR)	4 (3–5)	
Breed	Local	0	(0)
	Exotic	200	(100)
**Farms (Total)**		20	
Production method	Open field	0	(0)
	Pens	20	(100)
	Both	0	(0)
Annual production turnover	30–100	8	(40)
	101–300	8	(40)
	>300	3	(15)
	no data	1	(5)
Source of farm feed	Commercial feed	3	(15)
	Self-made feed	15	(75)
	Both	2	(10)
Use of antimicrobials in the last 12 months	Yes	20	(100)
	No	0	(0)
Purpose of the antimicrobials used	Treatment/Prophylaxis	20	(100)
	Added to farm feed	0	(0)

IQR—interquartile range.

**Table 2:** Antimicrobials (grouped in their different classes) used on the 20 Pig Farms in the Greater Accra Region of Ghana, from January to March 2022, and their respective WHO AWaRe classifications.

Name of Antimicrobial	AWaRe Classification	Number of Farms	
		n	(%)
Streptomycin/Gentamicin	Watch/Access	13	(65)
Penicillin/Amoxicillin	Access/Access	11	(55)
Oxytetracyline/Tetracycline/Doxycycline	Watch/Access/Access	9	(45)
Sulfonamide/Trimethoprim	Access/Access	6	(30)
Tylosin tartrate/Erythromycin	Not classfied in AWaRe/Watch	5	(25)
Enrofloxacin	Not classified in AWaRe	2	(10)
Colistin sulphate	Reserve	1	(5)

WHO Access, Watch, Reserve (AWaRe) classification of antibiotics for evaluation and monitoring of use in human health.

**Table 3:** Numbers and proportions of *Enterobacteriaceae* (*Escherichia coli, Klebsiella pneumoniae* and *Enterobacter* spp.) isolated from rectal swabs of healthy pigs in the Greater Accra Region of Ghana between January and March 2022.

Variable		Number	(%)
Rectal Swabs done		200	
*Enterobacteriaceae* present	Yes	197	(98.5)
	No	3	(1.5)
Categories of *Enterobacteriaceae*	*Escherichia coli* only	157	(79.7)
	*Enterobacter* spp. only	2	(1.0)
	*Klebsiella pneumoniae* only	0	(0)
	*Escherichia coli* AND *Enterobacter* spp.	35	(17.8)
	*Escherichia coli* AND *Klebsiella pneumoniae*	2	(1.0)
	*Escherichia coli* AND *Klebsiella pneumoniae* AND *Enterobacter* spp.	1	(0.5)

**Table 4:** Multi-drug Resistant *Enterobacteriaceae* isolated from healthy pigs in the Greater Accra Region of Ghana between January and March 2022.

Bacteria	Total	R3		R4		R5		R6	
	n	n	(%)	n	(%)	n	(%)	n	(%)
*Escherichia coli*	195	18	(9)	16	(8)	9	(5)	1	(0.5)
*Klebsiella pneumoniae*	3	0	(0)	0	(0)	0	(0)	0	(0)
*Enterobacter* spp.	38	1	(3)	1	(3)	0	(0)	0	(0)

Multi-drug resistance = resistance to three or more of the antibiotic classes: R3  =  resistant to three antimicrobial classes; R4  =  resistant to four antimicrobial classes; R5  =  resistant to five antimicrobial classes; R6  =  resistant to six antimicrobial classes.

**Table 5:** Profiles of the Multi-drug Resistant *Enterobacteriaceae* of Healthy Pigs in the Greater Accra Region of Ghana between January and March 2022.

Number of Resistant Classes	Resistance Profiles	Bacterial Isolates (n = 46)	
		n	(%)
3	TCY;AMC;AMP	6	(13.0)
	SXT;TCY;AMP	3	(6.5)
	SXT;TCY;CIP	1	(2.2)
	CAZ;AMC;AMP	3	(6.5)
	CAZ;GEN;AMC	1	(2.2)
	CAZ;TCY;AMP	2	(4.3)
	CAZ;TCY;AMC	1	(2.2)
	CAZ;TCY;ATM	1	(2.2)
	CAZ;CHL;TCY	1	(2.2)
4	GEN;ATM;AMC;AMP	1	(2.2)
	TCY;CIP;AMC;AMP	2	(4.3)
	TCY;GEN;AMC;AMP	2	(4.3)
	SXT;TCY;AMC;AMP	4	(8.7)
	SXT;TCY;ATM;AMC	1	(2.2)
	CHL;SXT;TCY;AMP	3	(6.5)
	CAZ;TCY;GEN;AMC	1	(2.2)
	CAZ;CHL;SXT;TCY	1	(2.2)
	CAZ;SXT;TCY;AMC	1	(2.2)
	CAZ;CHL;SXT;TCY	1	(2.2)
5	SXT;TCY;ATM;AMC;AMP	1	(2.2)
	SXT;TCY;GEN;AMC;AMP	1	(2.2)
	CHL;SXT;TCY;AMC;AMP	2	(4.3)
	CAZ;SXT;TCY;AMC;AMP	3	(6.5)
	CAZ;SXT;TCY;CIP;AMC	1	(2.2)
	CAZ;CHL;SXT;TCY;AMP	1	(2.2)
6	CAZ;SXT;TCY;GEN;AMC;AMP	1	(2.2)

AMP—ampicillin [penicillins]; AMC—amoxicillin/clavulanic acid [penicillins + beta-lactamase inhibitors]; CAZ—ceftazidime [cephalosporins]; SXT—trimethoprim/sulfamethoxazole [folate pathway inhibitors]; TCY—tetracycline [tetracyclines]; GEN—gentamicin [aminoglycosides]; CHL—chloramphenicol [phenicols]; ATM—aztreonam [monobactams]; CIP—ciprofloxacin [fluoroquinolones].

**Table 6:** Colistin resistance in *Enterobacteriaceae* of healthy pigs in the Greater Accra Region of Ghana between January and March 2022.

Bacteria	All isolates	Phenotypic Colistin Resistance		*mcr-1* Gene Detected	
	n	n	(%, 95% CI)	n	(%, 95% CI)
*Enterobacteriaceae*	236	18	(7.6, 95% CI 4.7–11.6)	10	(55.6, 95% CI 32.7–76.8)
*E. coli*	195	15	(7.7, 95% CI 4.5–12.1)	9	(60.0, 95% CI 34.5–81.9)
*Enterobacter* spp.	38	3	(7.9, 95% CI 2.0–20.0)	1	(33.3, 95% CI 1.7–86.8)

The *mcr-1* gene was only investigated in isolates showing phenotypic colistin resistance. None of the three isolates of *K. pneumoniae* showed phenotypic colistin resistance.

**Table 7:** Factors associated with MDR in *Enterobacteriaceae* of healthy pigs in the Greater Accra Region of Ghana between January and March 2022.

Variable	Pigs with *Enterobacteriaceae* n	Presence of MDR		Prevalence Ratio	(95% CI)
		n	(%)		
**Pig characteristics**					
Total pig population	197	46	(23)		
*Sex*					
Male	103	22	(21)	0.84	(0.5–1.5)
Female	94	24	(26)	1	
*Age of pigs in months*					
2–5 months	162	39	(24)	1	
6–9 months	35	7	(20)	0.83	(0.4–1.6)
**Farm characteristics**					
Annual pig production turnover *					
30–100	79	19	(24)	1	
101–300	78	20	(26)	1.06	(0.5–2.2)
>300	30	4	(13)	0.55	(0.2–1.9)
*Source of farm feed*					
Commercial	30	11	(37)	1.55	(0.8–3.2)
Self-made	148	35	(24)	1	
Both	19	0	(0)		

* With annual pig production turnover, there were missing data from one of the farms, and therefore the numbers do not add up to the total. MDR—multi-drug resistance (defined as resistance to three or more antimicrobial classes); CI—confidence interval.

**Table 8:** Factors associated with phenotypic colistin resistance in *Enterobacteriaceae* of healthy pigs in the Greater Accra Region of Ghana between January and March 2022.

Variable	Pigs with *Enterobacteriaceae*	Presence of Colistin Resistance		Prevalence Ratio	(95% CI)
	n	n	(%)		
**Pig characteristics**					
Total pig population	197	18	(9)		
*Sex*					
Male	103	12	(12)	1.83	(0.6–5.8)
Female	94	6	(6)	1	
*Age of pigs in months*					
2–5 months	162	17	(10)	1	
6–9 months	35	1	(3)	0.27	(0.1–2.0)
**Farm characteristics**					
Annual pig production turnover *					
30–100	79	6	(8)	1	
101–300	78	8	(10)	1.35	(0.4–4.1)
>300	30	1	(3)	0.44	(0.1–2.7)
*Source of farm feed*					
Commercial	30	16	(11)	0.62	(0.1–3.5)
Self-made	148	2	(7)	1	
Both	19	0	(0)		

* With annual pig production turnover, there were missing data from one of the farms, and therefore the numbers do not add up to the total. CI—confidence interval.

## Data Availability

The dataset used in this paper has been deposited at https://doi.org/10.6084/m9.figshare.20302461.v1 (accessed on 13 July 2022) and is available under a CC BY 4.0 license.
